# Characteristics of Physiological ^18^F-Fluoro-2-Deoxy-D-Glucose Uptake and Comparison Between Cats and Dogs With Positron Emission Tomography

**DOI:** 10.3389/fvets.2021.708237

**Published:** 2021-10-13

**Authors:** Yeon Chae, Taesik Yun, Yoonhoi Koo, Dohee Lee, Hakhyun Kim, Mhan-Pyo Yang, Byeong-Teck Kang

**Affiliations:** Laboratory of Veterinary Internal Medicine, College of Veterinary Medicine, Chungbuk National University, Cheongju, Chungbuk, South Korea

**Keywords:** canine, feline, 18F-FDG, FDG uptake, glucose metabolism, oncology, PET/CT, physiologic metabolism

## Abstract

This study aimed to identify the physiological 18F-fluoro-2-deoxy-D-glucose (FDG) uptake in cats using positron emission tomography/computed tomography (PET/CT) and determine its characteristics by comparing physiological differences with dogs. Seven healthy cats and six healthy beagle dogs were examined using FDG-PET/CT. Regions of interest (ROIs) were manually drawn over 41 detailed structures of 5 gross structures (brain, head and neck, musculoskeleton, thorax, and abdomen). The mean and maximum standard uptake values (SUVmean and SUVmax) were calculated for each ROI. Physiological variation was classified as having increased radiopharmaceutical activity with no evidence of abnormal clinical or radiological findings. The brain had the highest SUV, which was observed in the cerebellum of both cats (SUVmean: 4.90 ± 1.04, SUVmax: 6.04 ± 1.24) and dogs (SUVmean: 3.15 ± 0.57, SUVmax: 3.90 ± 0.74). Cats had a significantly higher intracranial uptake than dogs did (*P* < 0.01). In the digestive system, the SUVs of the duodenum and jejunum were significantly higher in dogs than in cats (*P* < 0.05). FDG uptake of the submandibular tip, tonsils, neck of the gallbladder, and caudal colliculus were physiologically increased in cats. This study demonstrates physiological FDG uptake in normal tissues, and the differences between cats and dogs were interpreted based on species-specificity. This information contributes to improving the accurate diagnosis of cancer in cats and will aid in understanding glucose metabolism in both cats and dogs.

## Introduction

^18^F-fluorodeoxy-2-deoxy-D-glucose (FDG) is similar to glucose because it enters the cell *via* a glucose transporter protein (GLUT) and is phosphorylated by hexokinase. However, FDG differs from glucose because it does not undergo glucose metabolism and can accumulate in the cell matrix. Positron emission tomography (PET) uses radiopharmaceuticals, such as FDG, to visualize glucose metabolism in a patient (1). FDG-PET can identify malignancy and the functional status of tissues by quantifying the standardized uptake value (SUV) (2). Therefore, the oncology field mainly uses FDG-PET/computed tomography (CT) to show tissue malignancy or metastasis to other organs (2).

In humans and dogs, physiological FDG uptake and normal ranges of SUV have been closely evaluated in each organ (3–5). However, in cats, there are only two studies on the normal distribution of FDG restricted to several organs, including the myocardium, liver, kidney, spleen, and colon, compared with humans and dogs (6), and on physiological variation (7); the normal range of SUV for each organ has not yet been measured. Tumors are the third leading cause of death in cats >5 years of age, and mortality increases with age. Approximately 50% of tumors cannot be identified on the body surface, and 80% of these are diagnosed as malignant, making early diagnosis and treatment difficult (8, 9).

This study aimed to clarify the physiological FDG uptake of 41 detailed structures of 5 gross structures in healthy cats and to understand the physiological variation compared with dogs.

## Materials and Methods

### Animals

Seven cats (four males and three females), weighing 5.69 ± 2.41 kg [mean ± standard deviation (SD)] and ranging from 3 to 5 years of age were recruited between May 1, 2020, and July 31, 2020. Six beagle dogs (two males and four females), weighing 8.52 ± 1.69 kg and ranging from 2 to 4 years of age were used in this study. All dogs and cats were healthy without a history of disease and had no abnormal signs on physical examination. They were tested for metabolic diseases using a complete blood count and serum chemistry profile. The experimental protocol was approved by the Institutional Animal Care and Use Committee (CBNUA-1413-20-01).

### Animal Preparation and Anesthesia

All animals fasted for 12 h before induction of anesthesia. Blood glucose was 122.70 ± 17.98 mg/dl in cats, and 90.33 ± 9.03 mg/dl in dogs. After intravenous catheter placement, anesthesia was induced with propofol (Provive, Myungmoon Pharm, Seoul, Republic of Korea) (cats: 6–8 mg/kg; dogs: 4-8 mg/kg). After endotracheal tube intubation, anesthesia was maintained with isoflurane (Terrell, Piramal Critical Care, Bethlehem, PA, USA) at 2.5-3.0% of the inspired volume during scanning in a circle rebreathing system.

During PET/CT examination, animals were positioned in sternal recumbency. Vital signs, including heart rate, oxygen saturation (SPO2), end-tidal CO2-concentration, and blood pressure were continuously monitored, and the animals were warmed with a warming pad (Equator; Surgi-Vet, Saint Paul, MN, USA).

### Image Analysis

The PET/CT system (Discovery-72 STE, General Electric Medical Systems, Waukesha, WI, 73 USA) was used for the FDG-PET scan. Each animal was administered 5.18–6.29 MBq/kg of FDG. The mean dosage of FDG administered intravenously in a slow bolus was 33.30 ± 11.84 MBq in cats and 52.91 ± 11.34 MBq in dogs, followed by 0.9% NaCl flushing. After injection of the radioisotope, a helical CT scan was performed throughout the whole body. Imaging parameters were 120 KV, 150 mAs, 8.75 mm/rotation, 0.875 pitch, 1.25 mm thickness, and 512 × 512 matrix. Whole-body PET images were obtained between 60 and 90 min after FDG administration in five to six bed positions (5 min each) depending on the size of the animal. Axial field of view (FOV) was 15.7 cm, trans-axial FOV was 70 cm, and axial sampling interval was 3.27 cm.

OsiriX MD v11.0 (Pixmeo Sarl, Geneva, Switzerland) was used to analyze the PET images. Regions of interest (ROIs) for each area were drawn manually over 41 detailed structures of 5 gross structures (brain: 6, head and neck: 10, musculoskeleton: 11, thorax: 4, abdomen: 10) (Tables 1–**4**; Supplementary Figures 1–5) and analyzed by three researchers (Y. C., T. Y., and B. K.). Most detailed structures were drawn on the transverse section; however, the aorta, caudal vena cava, spinal cord, and brain stem were drawn on the sagittal section of PET/CT fusion images.

**Table 1:** Mean and maximum SUVs of the brain in healthy cats and dogs.

**Regions**	**SUVmean**			**SUVmax**		
	**Cats**	**Dogs**	***P*-value**	**Cats**	**Dogs**	***P*-value**
Brain^*^	3.93 ± 0.89	2.61 ± 0.49	0.001^‡^	4.82 ± 1.11	3.28 ± 0.59	0.001^‡^
Frontal lobe	3.79 ± 0.76	2.76 ± 0.39	0.014^†^	4.43 ± 0.80	3.36 ± 0.59	0.022^**†**^
Parietal lobe	3.74 ± 0.77	2.58 ± 0.51	0.022^†^	4.27 ± 0.77	3.18 ± 0.56	0.022^**†**^
Temporal lobe	3.52 ± 0.64	2.52 ± 0.41	0.014^†^	4.21 ± 0.77	3.08 ± 0.54	0.022^**†**^
Occipital lobe	3.60 ± 0.70	2.20 ± 0.35	0.001^‡^	4.61 ± 0.88	2.95 ± 0.47	0.008^‡^
Cerebellum	4.90 ± 1.04	3.15 ± 0.57	0.002^‡^	6.04 ± 1.24	3.90 ± 0.74	0.002^‡^
Brain stem	4.02 ± 0.88	2.48 ± 0.16	0.001^‡^	5.36 ± 1.12	3.21 ± 0.23	0.005^‡^

*Results are presented as the mean ± SD*.

* *P < 0.001 for differences among detailed structures within the gross structure of cats and dogs (Kruskal-Wallis test)*.

† *P < 0.05, and*

‡ *P < 0.01 when comparing between cats and dogs (Mann-Whitney~U-test)*.

Increased radiopharmaceutical activity was estimated by comparing background variation, adjacent anatomy, and surrounding organ parenchyma. Physiological variation (**Table 5**; Supplementary Figure 6) was classified if increased radiopharmaceutical activity had no evidence of abnormal clinical or radiological findings.

The average tissue concentration of FDG (MBq/mL)/total injected dose (MBq)/body weight (g) was calculated for each ROI. SUVmean and SUVmax were calculated to quantitatively evaluate FDG. The same SUV upper threshold was used for the intensity of the PET-only and PET/CT fusion images for both cats and dogs. The red palette (thermal) lookup table was used in PET/CT fusion images.

### Statistical Analysis

Data were analyzed using GraphPad Prism 6 (GraphPad Software Inc., San Diego, CA, USA) and IBM SPSS Statistics version 22 (IBM, New York, USA). The mean and SDs were assessed using descriptive statistics. One-way ANOVA was used to compare the differences among the five gross structures. For small sample sizes, the Kruskal-Wallis test was performed to compare differences among the detailed structures. In a non-parametric assessment for a small sample size, the Mann-Whitney *U*-test was used to compare the SUVs of dogs and cats.

## Results

### Gross Structures

**Figure 1:**
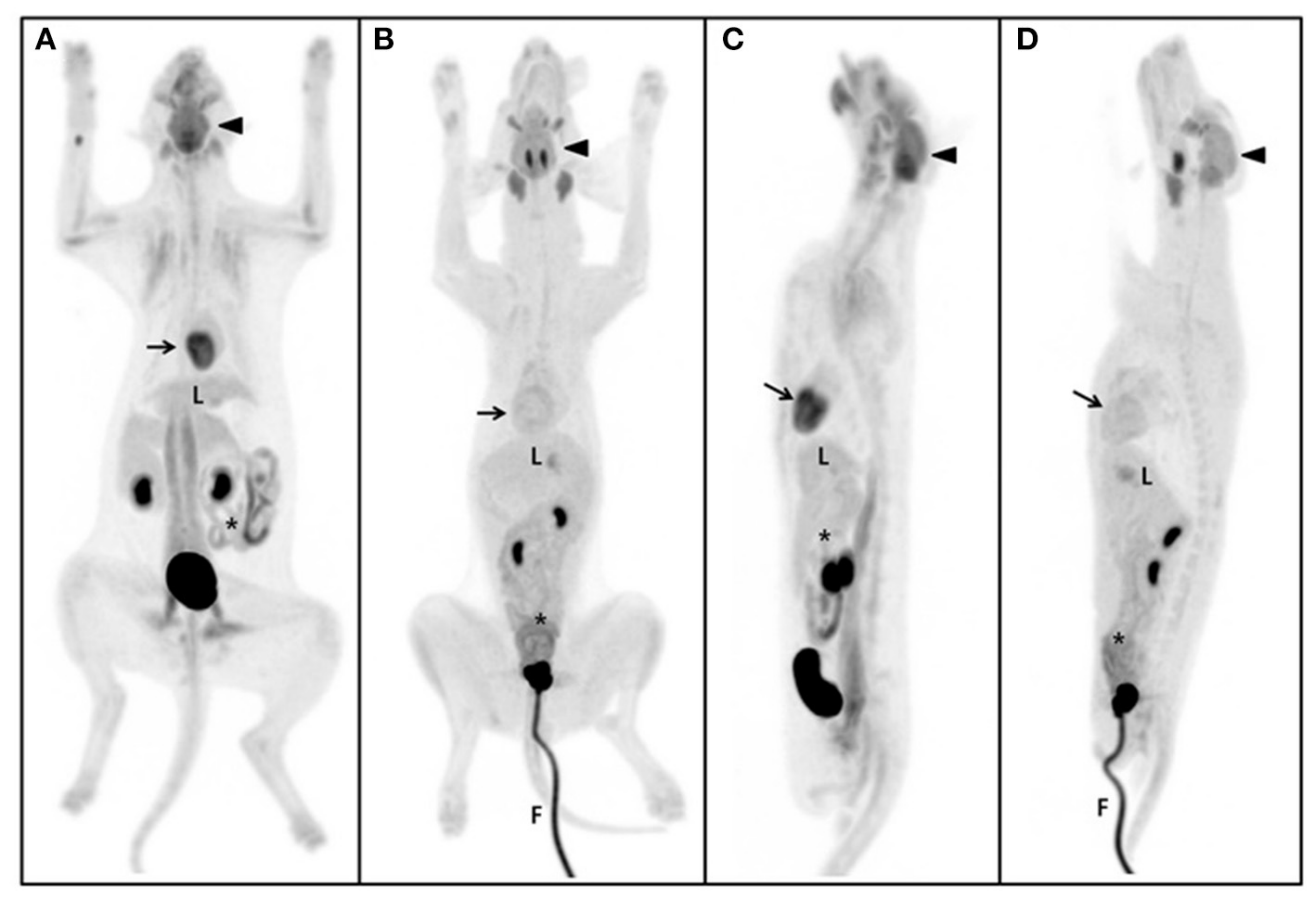
Maximum intensity projection (MIP) of the whole body. The same SUV upper threshold was used for the grayscale intensity of the PET-only images in both cats and dogs. Dorsal view of MIP in the cat **(A)** and dog **(B)**. Sagittal view of MIP in the cat **(C)** and dog **(D)**. The brain (arrow-head) had the highest SUV of the five gross structures and the cerebellum of the brain was the highest of the detailed structures in both cats and dogs. The second highest FDG uptake was detected in the myocardium (arrow) (excluding detailed brain structures), but not in dogs. The liver (L) is the major glucose synthesizing and storing organ; no significant differences were observed between cats and dogs. Parts of the intestine (asterisk), such as the duodenum and jejunum, were significantly higher in dogs than in cats. The gross musculoskeleton was significantly higher in cats than in dogs. F, foley catheter.

The estimated SUVmean and SUVmax in the 41 detailed structures are listed in Tables 1–4. Significant differences in FDG uptake were observed among the five gross structures (*P* < 0.001) (Figure 1). The brain had the highest FDG uptake in both cats (SUVmean: 3.93 ± 0.89, SUVmax: 4.82 ± 1.11) and dogs (SUVmean: 2.61 ± 0.49, SUVmax: 3.28 ± 0.59). The musculoskeleton had the lowest FDG uptake in dogs (SUVmean: 0.67 ± 0.30, SUVmax: 1.04 ± 0.40). Similarly, the musculoskeleton had the lowest SUVmean in cats (1.13 ± 0.63), whereas the abdomen had the lowest SUVmax (1.68 ± 0.66). FDG uptake in the brain, head and neck, and musculoskeleton was significantly higher in cats than in dogs (*P* < 0.05); however, the values in the abdomen were significantly higher in dogs (*P* < 0.01).

### Detailed Structures

Within each gross structure, SUVmean and SUVmax were significantly different among the 41 detailed structures (*P* < 0.001) (Figure 1). Among the 41 detailed structures, there were significant differences in the SUVmean of 24 structures and the SUVmax of 20 structures between cats and dogs (*P* < 0.05), and all of the SUVs in the detailed structures of the brain were significantly higher in cats than those in dogs (*P* < 0.05).

#### Brain

The FDG uptake of all detailed structures was significantly higher in cats than in dogs (*P* < 0.05) (Table 1). The highest uptake was identified in the cerebellum of cats (SUVmean: 4.90 ± 1.04, SUVmax: 6.04 ± 1.24) and dogs (SUVmean: 3.15 ± 0.57, SUVmax: 3.90 ± 0.74), whereas the lowest values were noted in the temporal lobe of cats (SUVmean: 3.52 ± 0.64, SUVmax: 4.21 ± 0.77) and the occipital lobe of dogs (SUVmean: 2.20 ± 0.35, SUVmax: 2.95 ± 0.47).

#### Head and Neck

The SUVs of the eyeball, lens, larynx, pharynx, tongue, and soft palate were significantly higher in cats than in dogs (*P* < 0.05) (Table 2). The highest SUVmean and SUVmax was observed in the zygomatic gland of cats (SUVmean: 2.69 ± 0.67, SUVmax: 3.55 ± 0.99) and dogs (SUVmean: 2.95 ± 0.40, SUVmax: 3.84 ± 0.80), whereas the lens had the lowest SUVmean in cats (0.88 ± 0.18) and dogs (0.66 ± 0.11). Furthermore, the lowest SUVmax was observed in the parotid gland of cats (1.45 ± 0.33) and the lens of dogs (0.99 ± 0.14).

**Table 2:** The mean and maximum SUVs of the head and neck in healthy cats and dogs.

**Regions**	**SUVmean**			**SUVmax**		
	**Cats**	**Dogs**	***P*-value**	**Cats**	**Dogs**	***P*-value**
Head and neck^*^	1.88 ± 0.69	1.56 ± 0.92	0.024^†^	2.54 ± 0.89	2.11 ± 1.17	0.021^†^
Eye ball	1.94 ± 0.32	1.02 ± 0.19	0.002^‡^	2.76 ± 0.38	1.42 ± 0.27	0.001^‡^
Lens	0.88 ± 0.18	0.66 ± 0.11	0.014^†^	1.48 ± 0.38	0.99 ± 0.14	0.008^‡^
Larynx	1.71 ± 0.33	1.01 ± 0.16	0.005^‡^	2.41 ± 0.60	1.65 ± 0.45	0.022^†^
Pharynx	1.85 ± 0.44	1.07 ± 0.23	0.002^‡^	2.50 ± 0.84	1.52 ± 0.26	0.035^†^
Tongue	2.05 ± 0.57	0.89 ± 0.15	0.001^‡^	2.82 ± 0.66	1.35 ± 0.38	0.005^‡^
Soft palate	2.11 ± 0.56	1.41 ± 0.50	0.022^†^	2.43 ± 0.59	1.67 ± 0.66	0.022^†^
Parotid gland	1.01 ± 0.17	1.09 ± 0.35	0.366	1.45 ± 0.33	1.37 ± 0.41	0.945
Sublingual gland	2.09 ± 0.55	2.56 ± 0.68	0.234	2.56 ± 0.67	3.57 ± 0.72	0.073
Mandibular gland	2.48 ± 0.51	2.94 ± 0.40	0.073	3.39 ± 0.74	3.74 ± 0.59	0.366
Zygomatic gland	2.69 ± 0.67	2.95 ± 0.54	0.534	3.55 ± 0.99	3.84 ± 0.80	0.731

*Results are presented as the mean ± SD*.

* *P < 0.001 for differences among detailed structures within the gross structure of cats and dogs (Kruskal-Wallis test)*.

† *P < 0.05, and*

‡ *P < 0.01 when comparing between cats and dogs (Mann-Whitney U-test)*.

#### Musculoskeleton

The SUVs of the vertebral body, spinal cord, cervical hypaxial muscle, brachial muscle, humerus, femur, psoas muscle, and lumbar hypaxial muscles were significantly higher in cats than in dogs (*P* < 0.05) (Table 3). In cats, the psoas muscle had the highest FDG uptake (SUVmean: 2.18 ± 0.84, SUVmax: 3.33 ± 1.24), whereas the lumbar epaxial muscle had the lowest uptake (SUVmean: 0.44 ± 0.17, SUVmax: 0.86 ± 0.30). The SUVs of dogs were highest in the spinal cord (SUVmean: 1.35 ± 0.20, SUVmax: 1.84 ± 0.31) and lowest in the femur (SUVmean: 0.47 ± 0.06, SUVmax: 0.71 ± 0.12).

**Table 3:** The mean and maximum SUVs of the musculoskeleton in healthy cats and dogs.

**Regions**	**SUVmean**			**SUVmax**		
	**Cats**	**Dogs**	***P*-value**	**Cats**	**Dogs**	***P*-value**
Musculoskeleton^*^	1.13 ± 0.63	0.67 ± 0.30	0.001^‡^	1.82 ± 1.07	1.04 ± 0.40	0.001^‡^
Vertebral body	1.18 ± 0.14	0.92 ± 0.16	0.022^†^	1.69 ± 0.33	1.35 ± 0.27	0.073
Spinal cord	1.73 ± 0.24	1.35 ± 0.20	0.008^‡^	2.34 ± 0.40	1.84 ± 0.31	0.035^†^
Cervical hypaxial muscle	1.15 ± 0.43	0.57 ± 0.20	0.005^‡^	2.14 ± 1.60	0.96 ± 0.24	0.001^‡^
Cervical epaxial muscle	0.71 ± 0.09	0.54 ± 0.15	0.051	1.25 ± 0.43	0.80 ± 0.21	0.051
Brachial muscle	1.09 ± 0.37	0.54 ± 0.19	0.014^†^	2.48 ± 1.21	0.90 ± 0.27	0.014^†^
Thigh muscle	0.62 ± 0.13	0.66 ± 0.25	0.945	1.10 ± 0.32	0.97 ± 0.32	0.534
Humerus	1.00 ± 0.37	0.50 ± 0.13	0.035^†^	1.44 ± 0.51	0.74 ± 0.17	0.035^†^
Femur	0.78 ± 0.15	0.47 ± 0.06	0.001^‡^	1.10 ± 0.27	0.71 ± 0.12	0.008^‡^
Psoas muscle	2.18 ± 0.84	0.70 ± 0.16	0.001^‡^	3.33 ± 1.24	1.27 ± 0.22	0.001^‡^
Lumbar hypaxial muscle	1.58 ± 0.75	0.58 ± 0.16	0.002^‡^	2.25 ± 1.11	1.00 ± 0.26	0.008^‡^
Lumbar epaxial muscle	0.44 ± 0.17	0.53 ± 0.17	0.295	0.86 ± 0.30	0.81 ± 0.27	0.731

*Results are presented as the mean ± SD*.

* *P < 0.001 for differences among detailed structures within the gross structure of cats and dogs (Kruskal-Wallis test)*.

† *P < 0.05, and*

‡ *P < 0.01 when comparing between cats and dogs (Mann-Whitney U-test)*.

#### Thorax

In most intrathoracic structures, SUVs did not significantly differ between cats and dogs (*P* > 0.05) (Table 4). Only the SUVmean of the lungs was significantly higher in cats than in dogs (*P* < 0.01). The myocardium had the highest FDG uptake in cats (SUVmean: 2.80 ± 0.58, SUVmax: 3.91 ± 1.19) and dogs (SUVmean: 1.94 ± 0.71, SUVmax: 2.58 ± 0.90). The lungs had the lowest uptake in dogs (SUVmean: 0.43 ± 0.06, SUVmax: 0.96 ± 0.16). In cats, the lung had the lowest SUVmean (0.71 ± 0.23) and the aorta the SUVmax (0.96 ± 0.20).

**Table 4:** The mean and maximum SUVs of the thorax and the abdomen in healthy cats and dogs.

**Regions**	**SUVmean**			**SUVmax**		
	**Cats**	**Dogs**	**P-value**	**Cats**	**Dogs**	**P-value**
Thorax^*^	1.26 ± 0.90	0.99 ± 0.65	0.215	1.69 ± 1.37	1.35 ± 0.78	0.303
Myocardium	2.80 ± 0.58	1.94 ± 0.71	0.126	3.91 ± 1.19	2.58 ± 0.90	0.082
Aorta	0.87 ± 0.17	0.98 ± 0.20	0.366	0.96 ± 0.20	1.14 ± 0.18	0.234
Caudal vena cava	0.88 ± 0.21	0.76 ± 0.18	0.366	1.02 ± 0.25	0.94 ± 0.20	0.945
Lung	0.71 ± 0.23	0.43 ± 0.06	0.001^‡^	1.12 ± 0.34	0.96 ± 0.16	0.589
Abdomen^*^	1.19 ± 0.43	1.46 ± 0.60	0.005^‡^	1.68 ± 0.66	2.08 ± 0.75	0.002^‡^
Gallbladder	0.73 ± 0.11	0.57 ± 0.20	0.138	1.35 ± 0.23	1.49 ± 0.62	0.945
Liver	1.63 ± 0.30	1.46 ± 0.27	0.234	2.05 ± 0.37	1.96 ± 0.40	0.836
Spleen	1.20 ± 0.23	1.16 ± 0.22	0.628	1.59 ± 0.24	1.56 ± 0.27	0.731
Pancreas	0.98 ± 0.36	1.27 ± 0.11	0.073	1.29 ± 0.46	1.60 ± 0.25	0.181
Kidney	1.48 ± 0.58	1.81 ± 0.24	0.534	2.35 ± 1.07	2.65 ± 0.38	0.731
Adrenal gland	1.08 ± 0.19	1.50 ± 0.34	0.035^†^	1.29 ± 0.24	1.62 ± 0.29	0.073
Stomach	1.11 ± 0.13	0.89 ± 0.25	0.181	1.51 ± 0.15	1.70 ± 0.36	0.445
Duodenum	0.90 ± 0.32	1.62 ± 0.18	0.002^‡^	1.37 ± 0.51	2.17 ± 0.20	0.014^**†**^
Jejunum	1.54 ± 0.35	2.41 ± 0.36	0.002^‡^	2.19 ± 0.92	3.35 ± 0.65	0.051
Colon	1.27 ± 0.61	1.90 ± 0.87	0.295	1.89 ± 0.71	2.67 ± 0.96	0.234

*Results are presented as the mean ± SD*.

* *P < 0.001 for differences among detailed structures within the gross structure of cats and dogs (Kruskal-Wallis test)*.

† *P < 0.05, and*

‡ *P < 0.01 when comparing between cats and dogs (Mann-Whitney U-test)*.

#### Abdomen

The SUVmean of the adrenal gland, duodenum, and jejunum and the SUVmax of the duodenum were significantly higher in dogs than in cats (*P* < 0.05) (Table 4). In cats, the liver (SUVmean: 1.63 ± 0.30) or kidney (SUVmax: 2.35 ± 1.07) had the highest uptake, whereas the jejunum had the highest values in dogs (SUVmean: 2.41 ± 0.36, SUVmax: 3.35 ± 0.65). The gallbladder had the lowest uptake in cats (SUVmean: 0.73 ± 0.11, SUVmax: 1.35 ± 0.23) and dogs (SUVmean: 0.57 ± 0.20, SUVmax: 1.49 ± 0.62).

### Physiological Variation

**Figure 2:**
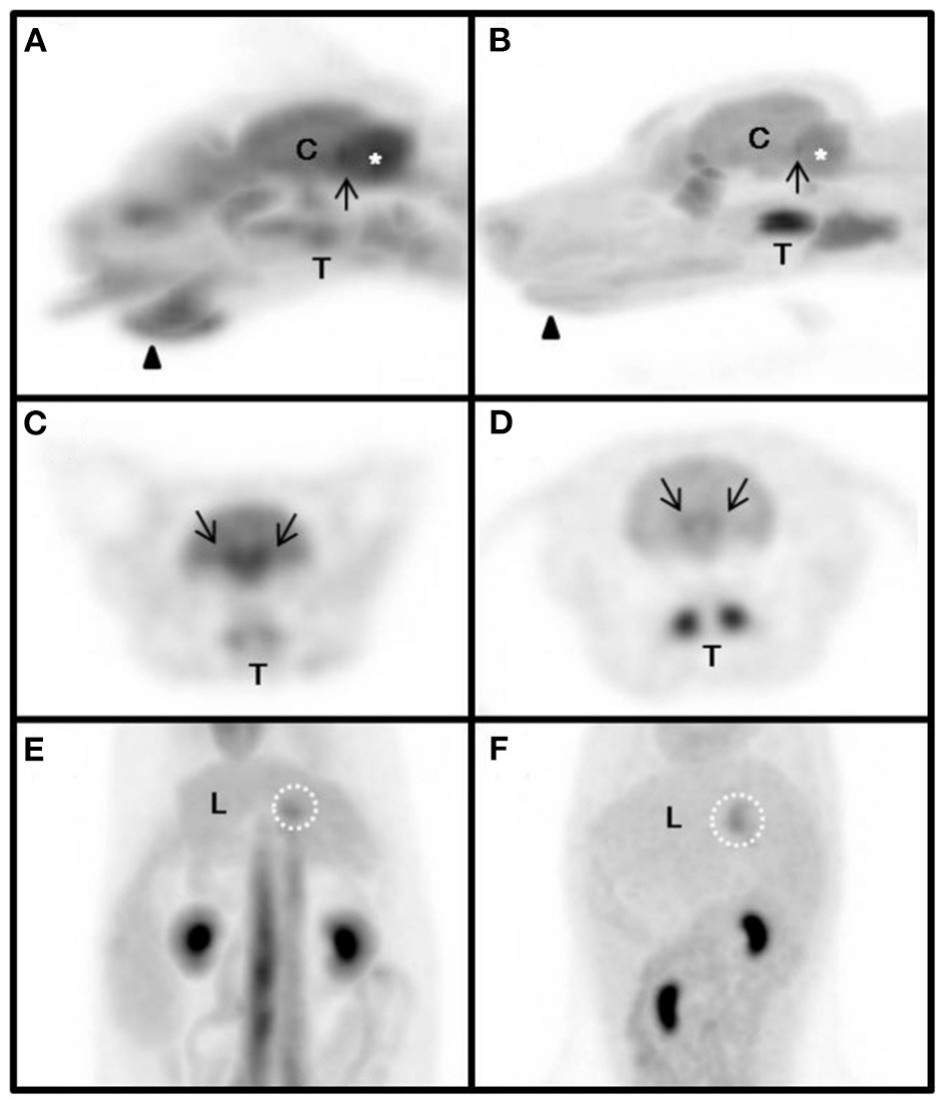
Magnified maximum intensity projection (MIP) of physiologic variants in the cat **(A,C,E)** and dog **(B,D,F)**. The same SUV upper threshold was used for the grayscale intensity of the PET-only images in both cats and dogs. Sagittal view of head **(A,B)**. Transverse view of head **(C,D)**. Dorsal view of abdomen **(E,F)**. arrow-head, submandibular tip; arrow, caudal colliculus; white dotted circle, gallbladder neck; asterisk, cerebellum; C, cerebrum; L, liver; T, tonsil.

Four structures including the submandibular tip, palatine tonsil, neck of the gallbladder, and caudal colliculus had physiological variation (Table 5) (Figure 2). The SUVs of the submandibular tip were significantly higher in cats than in dogs (*P* < 0.01), whereas the palatine tonsil had significantly lower uptake in cats (*P* < 0.05). FDG uptake was not different between cats and dogs in the neck of the gallbladder and the caudal colliculus of the midbrain (*P* > 0.05).

**Table 5:** The mean and maximum SUVs of physiologic variants in healthy cats and dogs.

**Regions**	**SUVmean**			**SUVmax**		
	**Cats**	**Dogs**	***P*-value**	**Cats**	**Dogs**	***P*-value**
Submandibular tip	3.01 ± 0.83	1.30 ± 0.20	0.001^†^	4.05 ± 0.85	1.58 ± 0.23	0.001^†^
Palatine tonsil	3.01 ± 1.26	4.84 ± 1.35	0.035^*^	3.81 ± 1.74	6.62 ± 1.34	0.022^*^
Gallbladder neck	1.80 ± 0.33	2.23 ± 0.40	0.073	2.41 ± 0.49	2.91 ± 0.58	0.073
Caudal colliculus	5.01 ± 0.90	3.81 ± 0.68	0.051	5.49 ± 0.98	4.41 ± 0.86	0.138

* *P < 0.05, and*

† *P < 0.01 when comparing between cats and dogs (Mann-Whitney U-test)*.

## Discussion

The physiological distribution of FDG and its significant differences among gross and detailed structures were demonstrated in cats and dogs. Previously, FDG uptake in the myocardium, liver, kidney, and colon was studied in cats and compared with the reported SUV in humans and dogs (6). However, there are no data describing the normal SUV in detailed structures in cats. Moreover, studies comparing SUV and blood glucose level (BGL) between cats and dogs have not yet been reported. In the present study, cats and dogs underwent PET/CT using the same protocol in the same environment. Therefore, the results could be compared more precisely between the two species because differences in PET/CT scanner and anesthesia protocol were excluded.

It is necessary to consider species-specific glucose metabolism to understand and discuss the normal glucose metabolic status in each region. Cats are carnivores and differ from dogs in glucose metabolism (10). Because cats have continuous activity of amino acid catabolic enzymes, the source of carbon skeletons for glucose production is constantly provided (10). Thus, cats have a carnivore-specific metabolism, unlike dogs, that maintains BGL through protein metabolism rather than conversion to lipid metabolism while fasting (10). In addition, plasma insulin is secreted in response to BGL and regulates glucose uptake into cells (10–12). This study showed a higher BGL in cats than in dogs; however, the plasma insulin concentration was not evaluated.

In this study, the brain had the highest SUV among five gross structures. The brain consumes the most glucose among the entire system as the default fuel; the main GLUT isoforms include GLUT1, which is highly expressed along the blood brain barrier (13). Because GLUT1 is insulin-independent, it ensures continuous influx of glucose into the brain regardless of BGL. Thus, the brain may have a higher SUV at a lower blood glucose status due to the characteristics of FDG that competes with glucose (13–16).

In general, the demand for glucose in the brain depends primarily on brain mass (15). The relative brain sizes and metabolic rates of cats are larger and higher than those of dogs, respectively (15), which may contribute to the higher FDG uptake in the brains of cats. Moreover, a previous study used the radioactive microsphere distribution method to show cerebral circulation in canines and felines (17). In both species, the pons, medulla, and cerebellum receive blood from the vertebral artery, but the thalamus and hypothalamus are supplied by the carotid and vertebral arteries (17). In cats, a greater portion of the vertebral arterial blood goes to the brain and is more specifically restricted to the pontomedullary and cerebellar structures, which receive approximately three times more microspheres in cats than in dogs (17). Therefore, higher FDG uptake in the cerebellum and brainstem can be influenced by the relatively larger blood flow to these areas in cats.

FDG uptake in the myocardium was the second highest in cats but not in dogs. In the fasting state, myocardial cells prefer to use lipid-derived fuel as the primary energy substrate by responding to BGL and converting glucose metabolism to lipid metabolism (18–20). Therefore, glucose metabolism in fasting dogs will be converted to lipid metabolism, and the myocardium may have lower glucose uptake than that without fasting. However, fasting cats maintain normal BGL through carnivore-specific metabolism, which continuously supplies gluconeogenic enzymes and sources for carbon skeletons (18–22). Therefore, myocardial FDG uptake in dogs may be lower than that in cats that maintain normal BGL while fasting; however, there were no significant differences between the two species because of the small sample size and the broad range of detected individual SUVs. Additionally, a previous study reported the SUVmean of myocardium in cats (3.58 ± 2.57) and compared it with that in dogs (1.80 ± 0.20) and non-fasted human (4.99 ± 2.90) (6). Similar to our study, the previous study mentioned that FDG uptake of myocardium in fasted cats resembles that of non-fasted human patients because of carnivore-specific metabolism in cats (6).

In humans, a significant inverse relationship was identified between the SUV in muscle and BGL at pre-PET scans (16). This finding could be explained by the competition between excessive endogenous blood glucose and FDG and the saturation of glucose transporters (16). However, this study showed significantly higher SUVs in the musculoskeleton in cats despite their higher BGL compared with dogs. Generally, the prominent muscle glucose transporter is GLUT4, which is insulin dependent (11, 23). In cats, mild to moderate elevation in serum glucose is a well-recognized physiological response to stress in clinical or research conditions, which is referred to as “stress hyperglycemia” (24). In addition, intravenous glucose tolerance tests of cats produce rapid glucose elevation, which would occur following insulin secretion (25). Thus, an unfamiliar hospital environment may contribute to the development of stress hyperglycemia. Because insulin could promote shifting glucose and FDG into muscle cells, relatively high uptake of FDG can be observed in the muscles of cats.

In contrast, the SUV in the duodenum and jejunum was significantly higher in dogs than in cats. As a carnivore, cats have decreased activities of pancreatic amylase and intestinal disaccharidase compared with omnivores, such as dogs (10). Therefore, cats may have decreased FDG uptake because of decreased activity of digestive glands.

In a previous human study, a positive correlation was found between pre-scan BGLs and SUVs in the liver (16). Additionally, a previous veterinary study reported that cats had the lowest SUV in the liver compared with dogs and humans because of lack of glucokinase, which phosphorylates glucose in hepatocytes (6). Contrary to this, the SUV in the liver did not differ significantly between cats and dogs in our study. In the liver, the bidirectional transporter GLUT2 is the major glucose transporter that regulates glucose fluxes based on the glucose diffusion gradient (10, 23). The liver is responsible for the regulation of blood glucose through gluconeogenesis and glycogenolysis in the fasting state (26) and efflux of glucose into the bloodstream via GLUT2 (27). In fasted dogs, the liver may produce and secrete glucose through both glycogenolysis and gluconeogenesis to maintain normal BGL; the status of glucose efflux by GLUT2 is superior to that of glucose influx in the liver. Therefore, similar to glucose, FDG uptake in the liver is decreased in fasting dogs. Cats may have no significant differences in FDG uptake in the liver compared with dogs, despite the lack of glucokinase activity.

In the bloodstream, SUV can be lower in cats than in dogs because of lower D-glucose transport and hexokinase activities of erythrocytes in cats (28). Nevertheless, the results of blood pools, such as the aorta and caudal vena cava, were not significantly different between cats and dogs. A previous human study reported that mediastinal blood pool SUVs have a significant positive correlation with pre-scan BGL because of GLUT1, which is the main erythrocyte GLUT and is not insulin-dependent (16, 29). If increased BGL due to stress hyperglycemia in cats affects to increase FDG uptake, SUV could increase and mask its lower SUV.

Increased radiopharmaceutical activity of the submandibular tip was detected in cats but not in dogs. Based on the anatomical and physiological specificity of cats, this region may be presumed to be a scent gland that is used to mark one's area, which does not exist in dogs in this region (30). In the laryngeal region, hyperactive tonsils were detected in cats. Hyperactive tonsils have been reported only in dogs and can be stimulated by an immune-induced status (7). Radiopharmaceutical activity increased in the neck of the gallbladder in cats, which was presumed to be caused by the accumulation of secreted bile juice containing circulating FDG, previously reported only in dogs (7). In a previous study, dogs showed the highest uptake in the caudal colliculus in the midbrain region (31). In this study, cats and dogs demonstrated the highest uptake in the caudal colliculus. The caudal colliculus can be stimulated by auditory stimuli, which may increase glucose and FDG consumption.

In feline oncology, oral squamous cell carcinoma (SCC) and fibrosarcoma have a high prevalence (32). In feline oral SCC, a previous study reported the SUVs (SUVmax 9.88 ± 5.33, range 2.9–24.9, average of SUVmean 5.39) of the primary tumor and thresholds of SUVmax (2.9–3.8, median 3.2) in hypermetabolic tumor regions (33). Also, in another previous study the SUV threshold of hypermetabolic tumor regions in SCC was 2.4–3.8 (median 3.2) (34). In general, the SUV > 2.5 suggests malignant tissue, but a wide range of SUVs has been reported for similar lesions (35). In this study, physiological variations in normal cats, such as those in the submandibular tip and palatine tonsil, were located around oral structures, which can be associated with oral SCC. Moreover, the SUV of these structures was > 3.0 and overlapped with the range of previously reported results in oncologic patients. Thus, the interpretation of SUVs around oral structures should be assessed carefully to avoid tumor misdiagnosis. A previous study on dogs with limb lameness reported the SUVs of regions with (SUVmax > 1.0) or without (SUVmax < 1.0) pathologies (36). Also, previously reported SUVmax of normal skeletal muscles in dogs was ~1.0 (4, 5), and these findings are consistent with those of the present study. As a diagnostic modality, FDG PET-CT could be valuable in detecting soft tissue abnormalities, which cannot be diagnosed by conventional means (36). However, in the present study, most of the SUVmax of skeletal muscles in normal cats were higher than 1.0. The SUV of a pathologic lesion in cats may differ from that in dogs because of different physiologies between the species. Therefore, the SUV results in normal cats in the present study could be useful in diagnosing musculoskeletal abnormalities, but careful assessment is needed due to the limited reports of abnormalities.

Compared to previously reported SUVs of normal cats (6) and dogs (5), there are mild differences, but direct comparison is difficult because SUVs could be affected by biological (e.g., BGL, FDG distribution and clearance, etc.) and technical factors (e.g., acquisition parameters, time frame duration, etc.) (37). Therefore, careful interpretation of the results in clinical cases is needed, and this study may be useful in assessment of clinical data.

This study has some limitations. First, the sample size was small; further studies with larger sample sizes may improve reliability. Second, the animals were anesthetized to restrict their movement to ensure accurate results. Anesthetics may influence glucose metabolism in patients; however, it is necessary for PET/CT in the veterinary field. Third, oncologic patient data were not included in this study. To be applicable in the oncologic field, it is important to identify accurate differences between healthy and abnormal tissues. Lastly, this study did not evaluate the hormones that affect glucose metabolism. Additional hormonal studies will be helpful in understanding and discussing species-specific metabolism.

In conclusion, this study demonstrated physiological FDG uptake in normal tissues throughout the body, and differences between cats and dogs were interpreted based on species-specificity. This study will contribute to improved diagnosis of malignancy using PET/CT and understanding of glucose metabolism in both cats and dogs.

## Data Availability Statement

The raw data supporting the conclusions of this article will be made available by the authors, without undue reservation.

## Ethics Statement

The animal study was reviewed and approved by Institutional Animal Care and Use Committee (CBNUA-1413-20-01). Written informed consent was obtained from the owners for the participation of their animals in this study.

## Author Contributions

YC, TY, YK, and DL contributed to management of the cats and dogs. YC and TY wrote the first draft of the manuscript. HK, M-PY, and B-TK participated in the revision of the manuscript. All authors read, commented on, and approved the final manuscript. All authors contributed to the article and approved the submitted version.

## Funding

This work was supported by the National Research Foundation of Korea (NRF) grant funded by the Korea government (MSIT) (No. 2021R1A2C1012058).

## Conflict of Interest

The authors declare that the research was conducted in the absence of any commercial or financial relationships that could be construed as a potential conflict of interest.

## Publisher's Note

All claims expressed in this article are solely those of the authors and do not necessarily represent those of their affiliated organizations, or those of the publisher, the editors and the reviewers. Any product that may be evaluated in this article, or claim that may be made by its manufacturer, is not guaranteed or endorsed by the publisher.

## Abbreviations

BGL: blood glucose level
FDG: 18F-fluoro-2-deoxy-D-glucose
FOV: field of view
GLUT: glucose transporter protein
PET/CT: positron emission tomography/computed tomography
ROI: region of interest
SCC: squamous cell carcinoma
SD: standard deviation
SUV: standard uptake value.
