# Supplementary material for: Characteristics of Physiological 18F-Fluoro-2-Deoxy-D-Glucose Uptake and Comparison Between Cats and Dogs With Positron Emission Tomography
Source: Front Vet Sci. 2021 Oct 13;8:708237. doi: 10.3389/fvets.2021.708237 (PMC8548631; doi:10.3389/fvets.2021.708237)
Supplement: Supplementary file 1 [file Data_Sheet_1.Pdf]

Supplementary Material

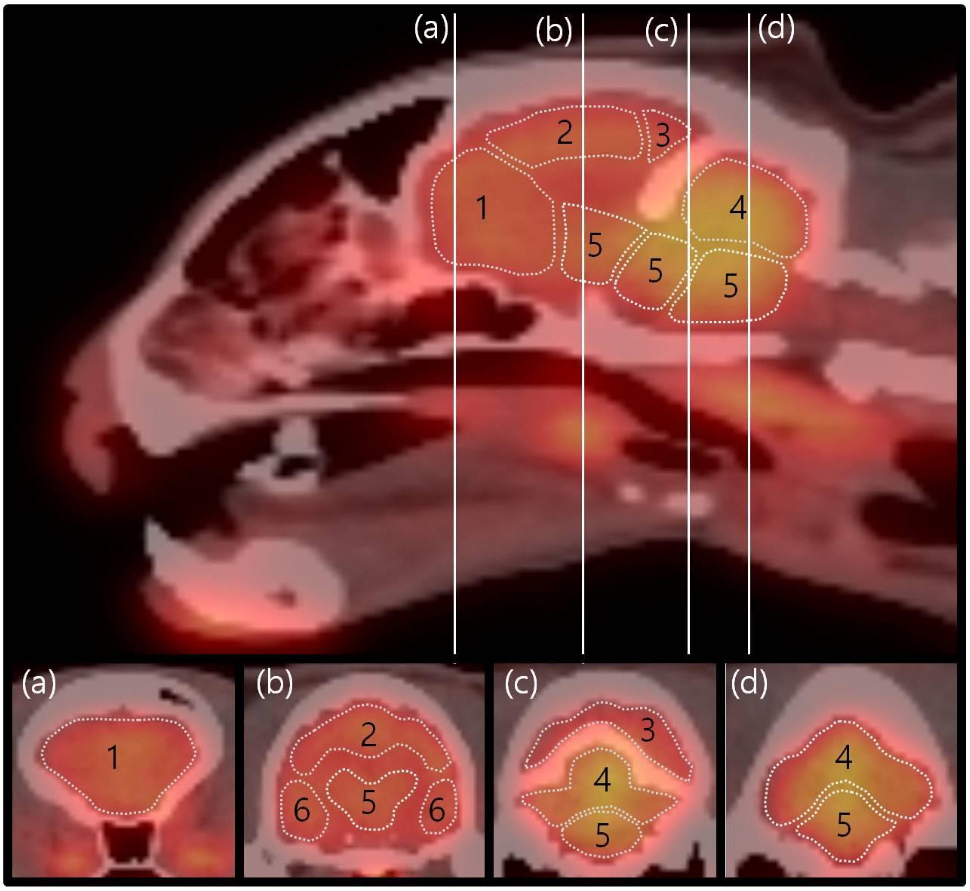

Supplementary Figure 1. Regions of interest (ROIs) of five detailed structures in the brain of the cat. White dotted lines are examples of ROIs assessed in this study. 1. frontal lobe, 2. parietal lobe, 3. occipital lobe, 4. cerebellum, 5. brain stem, 6. temporal lobe .

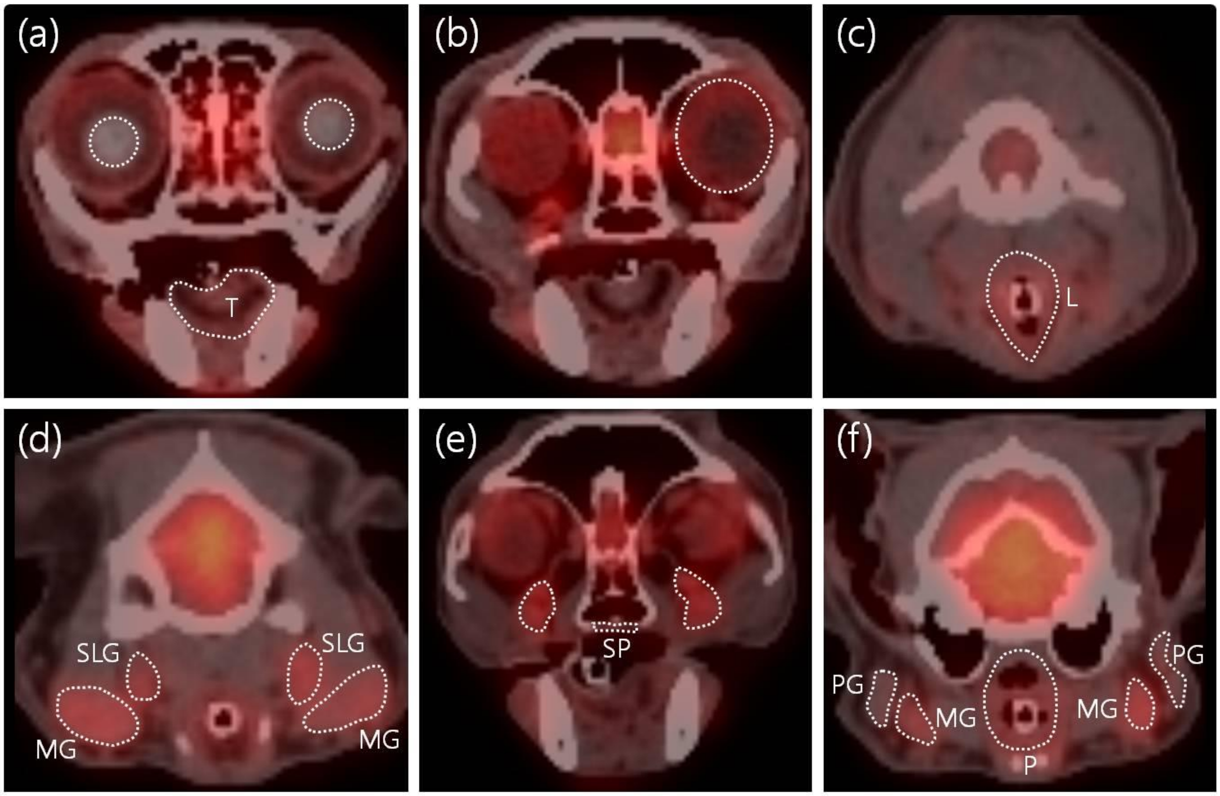

Supplementary Figure 2. Regions of interest (ROIs) of 10 detailed structures in the head and neck of the cat. White dotted lines are examples of ROIs assessed in this study. (a) Lens and tongue. (b) Eye ball without lens. (c) Larynx. (d) Mandibular salivary gland and sublingual salivary gland. (e) Zygomatic salivary gland and soft palate. (f) Parotid gland and pharynx. L, larynx; MG, mandibular salivary gland; P, pharynx; PG, parotid gland; SLG, sublingual salivary gland; SP, soft palate; T, tongue.

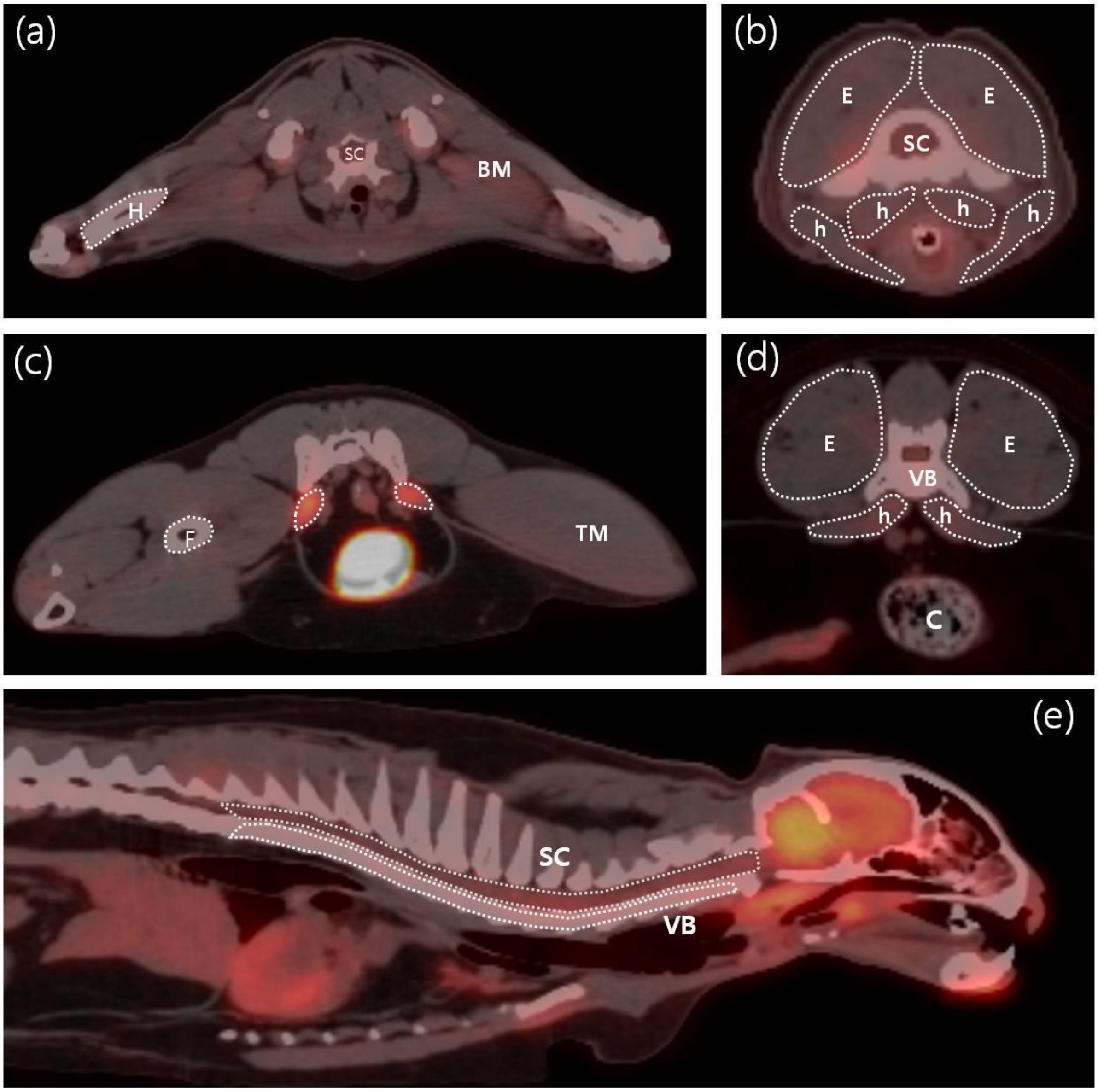

Supplementary Figure 3. Regions of interest (ROIs) of 11 detailed structures in the musculoskeleton of the cat. White dotted lines are examples of ROIs assessed in this study. (a) Brachial muscle and humerus. (b) Epaxial and hypaxial muscle of cervix. (c) Thigh muscle, psoas muscle, and femur. (d) Epaxial and hypaxial muscle of lumbar. (e) Spinal cord and vertebral body. BM, brachial muscle; C, colon; E, epaxial; F, femur; H, humerus; h, hypaxial; SC, spinal cord; VB, vertebral body.

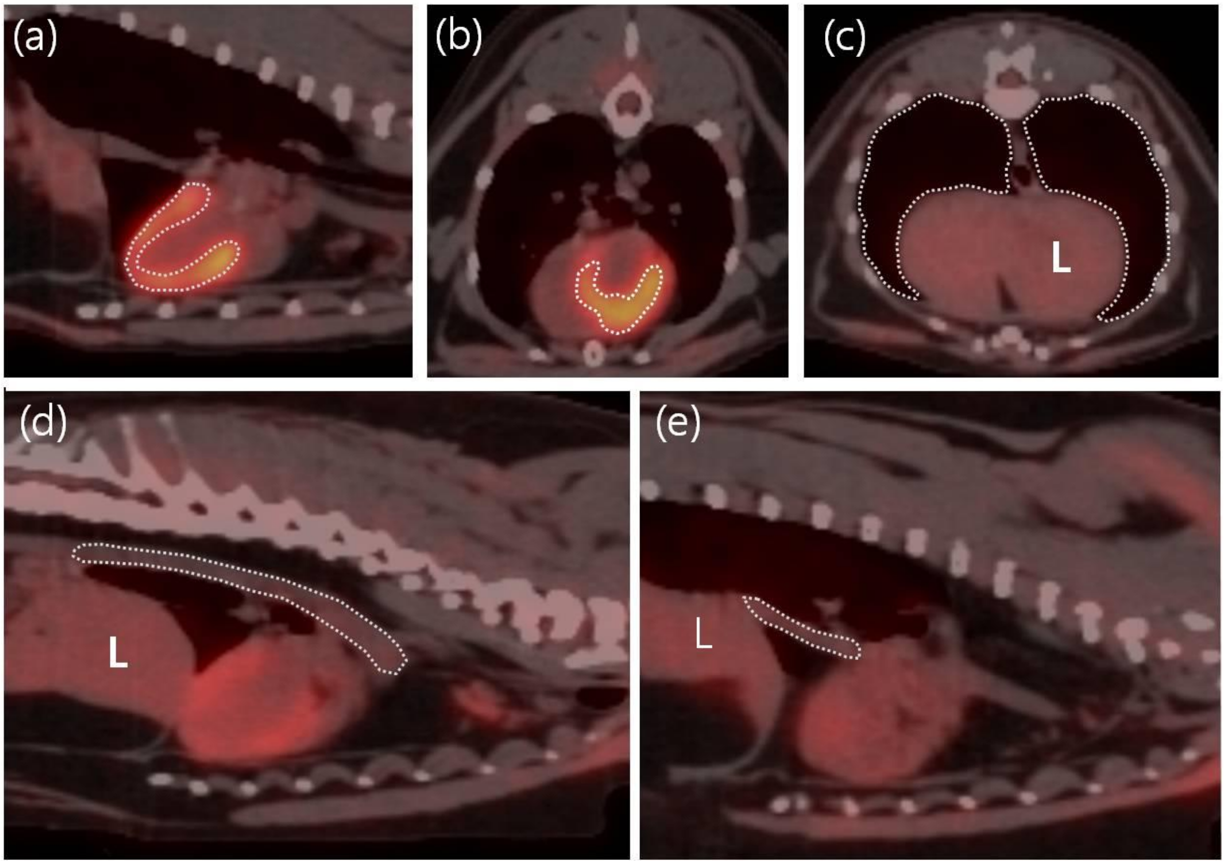

Supplementary Figure 4. Regions of interest (ROIs) of four detailed structures in the thorax of the cat. White dotted lines are examples of ROIs assessed in this study. (a) Myocardium in the sagittal view. (b) Myocardium, (c) lung, (d) aorta, and (e) caudal vena cava on the sagittal view. L, liver.

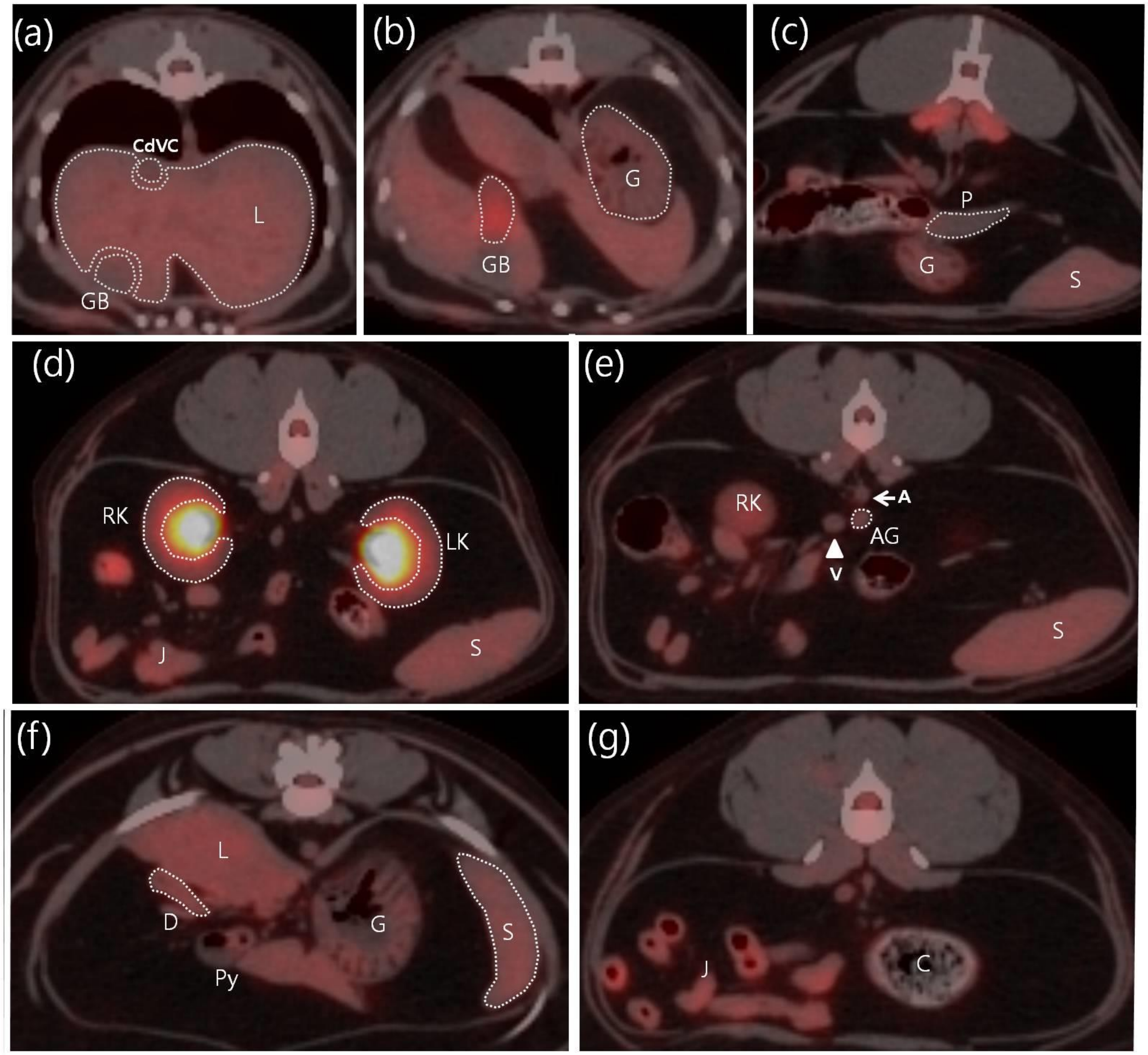

Supplementary Figure 5. Regions of interest (ROIs) of 10 detailed structures in the abdomen of the cat. White dotted lines are examples of ROIs assessed in this study. (a) Liver and gallbladder. (b) Stomach and gallbladder. (c) Pancreas. (d) Left and right kidney. (e) Adrenal gland. (f) Duodenum and spleen. (g) Jejunum and colon. A, aorta; AG, adrenal gland; C, colon; CdVC, caudal vena cava; D, duodenum; G, stomach; GB, gallbladder; J, jejunum; RK, right kidney; LK, left kidney; L, liver; P, pancreas; Py, pylorus; S, spleen; V, portal vein.

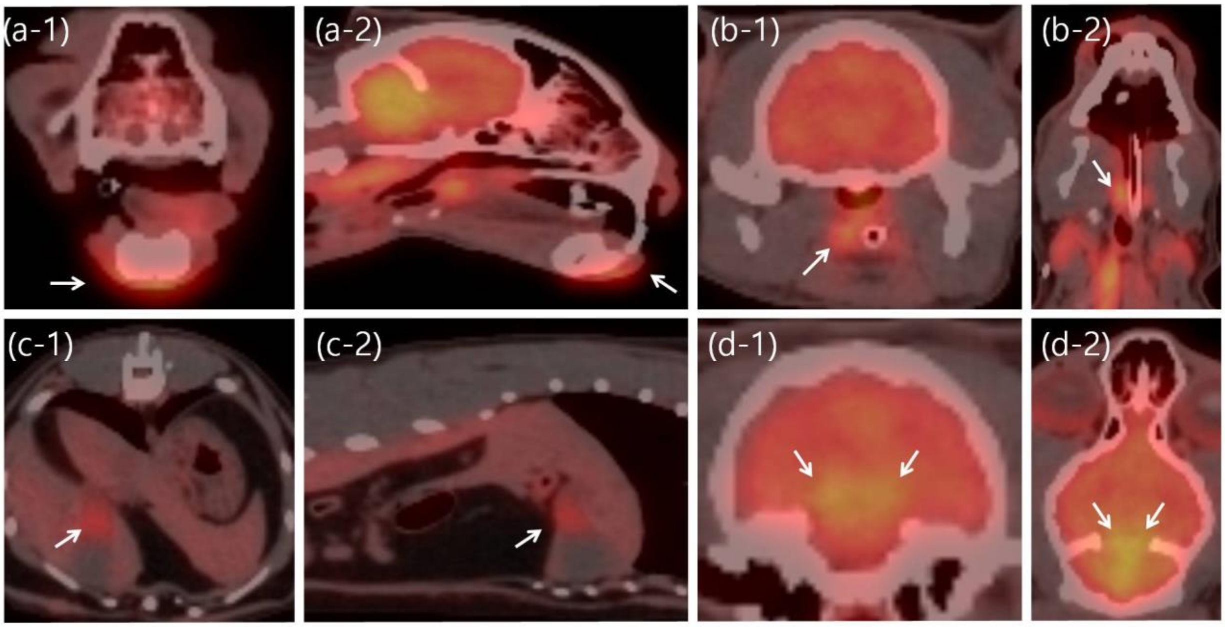

Supplementary Figure 6. Regions with increased physiological FDG uptake in four detailed structures of the cat. Submandibular tip in the transverse view (a-1) and sagittal view (a-2). Palatine tonsil in the transverse view (b-1) and coronal view (b-2). Gallbladder neck in the transverse view (c-1) and sagittal view (c-2). Caudal colliculus in the transverse view (d-1) and coronal view (d-2).
